# Persistent and severe hypotension during radical transabdominal ovarian cancer surgery: A case report

**DOI:** 10.1097/MD.0000000000040751

**Published:** 2024-12-06

**Authors:** Xinyan He, Hui Liu

**Affiliations:** a Department of Anesthesiology, West China Second University Hospital, Sichuan University, Key Laboratory of Birth Defects and Related Diseases of Women and Children, Sichuan University, Ministry of Education, Chengdu, Sichuan, China; b West China School of Medicine, Sichuan University, Chengdu, China.

**Keywords:** case report, hypoalbuminemia, ovarian cancer surgery, persistent hypotension

## Abstract

**Rationale:**

In radical surgery for ovarian cancer (OC), hypotension that is difficult to correct is usually rare unless there is significant blood loss. We recently encountered a patient who developed persistent and severe hypotension during radical transabdominal OC surgery.

**Patient concerns:**

A patient was 52 years old with a history of hypertension and well-controlled preoperative blood pressure (BP). A total of 2000 mL of ascites was drained and blood loss was 300 mL when the operation proceeded to 5.5 hours. The patient’s cardiopulmonary function and blood gas analysis showed no significant abnormalities.

**Diagnoses:**

persistent and uncorrectable hypotension.

**Interventions:**

There was no significant edema in the patient’s head or face, nor did the surgeon observe noticeable edema in her intestinal walls or other organs. No oozing was seen at the surgical site. Fluid resuscitation and vasopressor administration were continued. As BP control further deteriorated, blood counts, coagulation, and biochemical electrolyte analyses revealed severe hypoalbuminemia (13.5 g/L) and coagulation dysfunction.

**Outcomes:**

After intravenous human serum albumin (HSA) and fresh frozen plasma therapy, her hypoalbuminemia and coagulation were gradually corrected.

**Lessons:**

Based on this case, we suggest that in OC patients experiencing mild intraoperative bleeding and minimal heart rate variation but persistent refractory hypotension, hypoalbuminemia should be considered even if preoperative biochemical tests (including serum albumin levels) are normal. Confirming hypoalbuminemia warrants HSA administration to alleviate hypovolemic shock symptoms. Additionally, it is important to be cautious of potential coagulation issues with albumin use, possibly requiring plasma infusion to address coagulopathy.

**Key Points**

- The patient developed hypotension incompatible with bleeding during OC surgery.
- Hypoalbuminemia was identified as the cause of hypotension.
- Administration of HSA to patients with coagulation disorders worsened coagulation.

## 1. Introduction

Ovarian cancer (OC) often presents insidiously, with most patients diagnosed after intraperitoneal metastasis has occurred. Cytoreductive surgery is essential for patients with advanced OC to reduce tumor burden, improve clinical symptoms, and extend survival.^[1]^ Due to the complexity and invasiveness of the procedure, significant intraoperative bleeding is common. Typical manifestations of hypovolemic hypotension involve reflex tachycardia, which is usually corrected by blood and fluid replenishment. However, we recently encountered a unique case of advanced OC surgery where preoperative tests, except for slight chronic inflammation observed in the lungs on chest computed tomography (CT) and ascites on abdominal CT, showed no abnormalities. Although the surgery lasted 8 hours with an estimated blood loss of only 300 mL, the patient displayed stable heart rate (HR) and persistent severe hypotension, with increasing insensitivity to vasopressors as the procedure progressed.

## 2. Case presentation

A 52-year-old female patient, 165 cm in height and 70 kg in weight, was diagnosed with OC and advised to undergo radical transabdominal surgery. She had a history of hypertension managed with nifedipine sustained-release tablets, maintaining blood pressure (BP) between 120 to 130/60 to 80 mm Hg. Preoperative tests (including blood count, coagulation time, biochemical, electrolyte, and electrocardiogram tests; Table 1) were normal, with only a small amount of chronic inflammation noted in the lungs on chest CT and significant ascites on abdominal CT. Thirty minutes before surgery, a standard dose of antibiotics was administered. Anesthesia was induced via rapid sequence, maintained with 2.0% to 3.0% sevoflurane in 50% oxygen. BP was continuously monitored through invasive arterial puncture, and initial vital signs remained stable, with a HR of 83 to 87 bpm, BP at 100 to 130/60 to 80 mm Hg, and SPO_2_ at 100%. After the first 30 minutes, approximately 2000 mL of ascites was drained, with BP fluctuations stabilized by ephedrine administration.

**Table 1:** Blood count, coagulation, and biochemistry results.

	HB(g/L)	Hematocrit (%)	WBC (10^9^/L)	PLT (10^9^/L)	Serum albumin (g/L)	PT (s)	APTT (s)	Fn (mg/dL)	Fg (mmol/L)
Before surgery	139	44.5	9.5	325	44.8	11.3	26.8	476	5.72
Surgery was performed in 6.5 h	96	31.5	15.1	274	13.5	16.8	51.7	139	13.4
Admission to ICU	102	32	13.6	281	28.7	17.6	98.6	139	15.8

APTT = activated partial thromboplastin time, Fg = fasting glucose, Fn = fibrinogen, HB = hemoglobin, ICU = intensive care unit, PLT = platelet, PT = prothrombin time, WBC = white blood cell.

**Figure 1.**
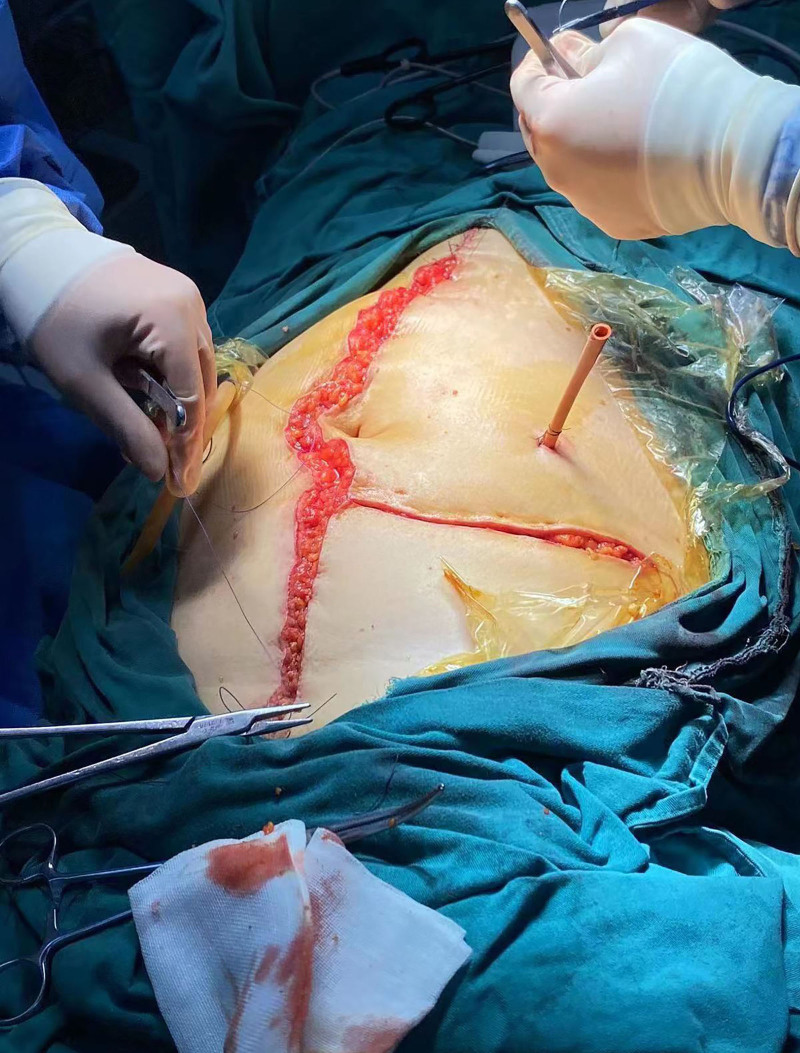
The scope of this surgical incision: superior to the subxiphoid process and inferior to above the pubic symphysis.

At 2.5 hours into the surgery, blood loss was approximately 100 mL, urine output was 100 mL, BP decreased to 80 to 90/40 to 50 mm Hg, and HR rose to 95 bpm with SPO_2_ between 98% and 100%. Lung sounds were clear and symmetric. Gastrointestinal and biliary surgeons rotated in to excise cancerous abdominal metastases. The incision extended from the xiphoid to the pubic symphysis (Fig. 1). Although the fluid balance was stable, latent hemorrhage and potential allergic reactions to antibiotic administration prompted us to accelerate fluid administration, administer dexamethasone 10 mg, and maintain continuous norepinephrine infusion at 0.03 to 0.1 µg/kg/minute. After intervention, BP rose to 90 to 100/50 to 60 mm Hg, and HR ranged between 80 and 90 bpm.

At 5.5 hours, the patient’s position was adjusted to head-down to facilitate excision of metastatic lesions near the liver and right diaphragm. BP suddenly dropped to 70 to 80/50 mm Hg, with SPO_2_ falling to 86%. With increased norepinephrine infusion, BP gradually improved to 80 to 85/50 to 53 mm Hg. A rapid fluid balance was conducted: 200 mL blood loss, 400 mL urine output, 2500 mL crystalloid, and 500 mL colloid had been administered. Based on preoperative fasting, intraoperative fluid loss, and administered fluids, we estimated the patient’s fluid balance as even. No allergic hives or rashes were visible. A possible pleural puncture was suspected but ruled out by inflating the patient’s lungs following diaphragmatic suturing, which confirmed no air leak into the right pleural cavity. After returning the patient to the supine position and recalibrating the arterial sensor, blood pressure was barely maintained at 90 to 95/50 to 55 mm Hg. We initially suspected orthostatic hypotension but remained vigilant.

Initially, we continued to increase the administration of crystalloid fluids; however, the patient’s BP did not improve. We suspected that the infused fluids were not effectively remaining in the vascular compartment. The most common reason for this phenomenon is a decrease in plasma colloid osmotic pressure, which can cause fluid to shift from the vascular space into the tissues, leading us to suspect that the patient might be experiencing hypoalbuminemia. We immediately verified the patient’s preoperative serum albumin level from the day before surgery, which was 44.8 g/L (normal range is typically 35 to 55 g/L). Subsequently, we did not observe any significant edema in the patient’s head and neck under the sterile surgical drapes, and the surgeon reported no noticeable exudate or edema at the surgical site. Based on this, we temporarily dismissed the suspicion of hypoalbuminemia. Next, we performed a concurrent ultrasound examination to exclude heart failure and pulmonary edema. Finally, to rule out a delayed allergic reaction due to antibiotic administration, we infused the patient with 1 g of calcium gluconate intravenously, which could help alleviate any latent allergic responses and prevent myocardial contractility reduction due to hypocalcemia. However, after 1 hour of treatment, there was still no significant improvement in the patient’s BP.

**Figure 2.**
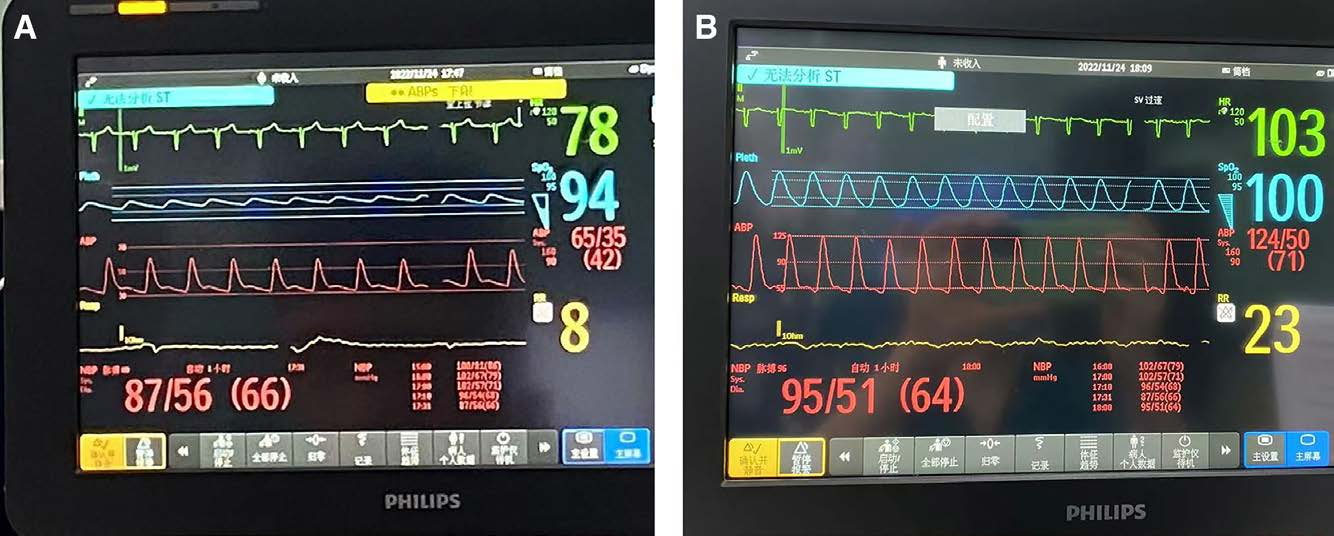
Comparison of vital signs before and after epinephrine administration. (A) Before epinephrine administration; (B) after epinephrine administration.

At 6.5 hours into the surgery, while administering norepinephrine at high doses, the patient’s BP suddenly dropped to 65/35 mm Hg (Fig. 2). We noted a rapid decrease in the patient’s response to norepinephrine, prompting us to switch to a rapid intravenous bolus of epinephrine. After administering 0.1 mg of epinephrine, the BP increased to 124/50 mm Hg (Fig. 2). Subsequently, we initiated a continuous infusion of epinephrine at a rate of 0.1 µg/kg/minute to maintain arterial pressure between 90 to 100/40 to 50 mm Hg. Due to the persistently low BP, the anesthesiologist was unable to successfully perform arterial blood gas analysis from the peripheral vein. We then requested laboratory staff to assist us in drawing peripheral blood from the patient in the operating room for urgent testing of the blood count, coagulation function, and biochemical markers (which included the patient’s protein levels and various electrolytes). Concurrently, we inserted a central venous catheter into the right internal jugular vein to measure central venous pressure and guide fluid administration. Thirty minutes later, the patient’s laboratory results (Table 1) indicated severe hypoalbuminemia and deteriorating coagulation function. We immediately began administering 60 g of human serum albumin (HSA) until the end of the surgery, and the patient’s BP gradually increased to 100 to 130/60 to 80 mm Hg. The surgery lasted a total of 8 hours, with an estimated blood loss of approximately 300 mL and urine output of 750 mL. A total of 8000 mL of crystalloid and 1500 mL of colloid were infused. After providing adequate postoperative analgesia, the patient was transferred to the intensive care unit (ICU) with an endotracheal tube for further treatment.

In the ICU, moist crackles were audible in both lungs. The ICU physician was able to maintain the BP at 110/80 mm Hg with norepinephrine at a dose of 0.1 µg/kg/minute. Subsequently, the ICU doctor performed a blood test for the patient, with results as shown in Table 1: the patient still exhibited persistent hypoalbuminemia and coagulation abnormalities. The ICU physician administered 600 mL of fresh frozen plasma to correct the coagulation issue and continued to infuse albumin to address the hypoalbuminemia. On postoperative day 1, the patient’s endotracheal tube was removed. By postoperative day 5, the serum albumin level had returned to 36.5 g/L, and the patient was discharged on postoperative day 8.

## 3. Discussion

According to the Common Terminology Criteria for Adverse Events version 5.0 by the US Department of Health and Human Services, hypoalbuminemia is graded in 5 levels (Table 2), with a normal albumin threshold set at 30 g/L. The patient’s serum albumin on the day before surgery was 44.8 g/L, within the normal range.^[2]^ However, the intraoperative albumin concentration dropped significantly to 13.5 g/L, coupled with critical circulatory instability and an immediate need for resuscitation, meeting criteria for grade 4 hypoalbuminemia.^[2]^ We identified several contributing factors: substantial protein loss through exudative ascites. Immune activation from surgical stress, releasing inflammatory cytokines like TNF-α, IL-2, IL-6, and VEGF, increasing vascular permeability and albumin leakage.^[3,4]^ Reduced albumin synthesis under surgical stress, favoring acute-phase proteins like C-reactive protein.^[5]^ Activation of the hypothalamic-pituitary-adrenal axis, reducing synthesis hormones and promoting protein catabolism.^[6]^ Extensive pelvic and aortic lymphadenectomy potentially limiting lymphatic protein reabsorption. Extensive use of crystalloids and colloids resulting in dilutional hypoalbuminemia.^[7]^

**Table 2:** Hypoalbuminemia: a disorder characterized by laboratory test results that indicate a low concentration of albumin in the blood.

Grade	Diagnostic criteria
1	<LLN - 3 g/dL; <LLN - 30 g/L
2	<3–2 g/dL; <30–20 g/L
3	<2 g/dL; <20 g/L
4	Life-threatening consequences; urgent intervention indicated
5	Death

From CTCAE 5.0.

CTCAE = Common Terminology Criteria for Adverse Events, LLN = lower limit of normal.

Reflecting on the diagnostic delay, the patient’s normal preoperative blood results and subtle signs hindered early identification of hypoalbuminemia. Hypoalbuminemia lowers plasma oncotic pressure, resulting in intravascular fluid shifts, reduced effective circulating volume, and hypovolemic shock, often indicated by edema in the limbs, eyelids, and abdomen. However, this patient showed no marked signs of such edema in the eyes, skin, or intestines, and even ultrasound ruled out pulmonary edema. We surmise that extensive incisional area and evaporative loss hindered visible symptoms of hypoalbuminemia.

Additionally, several other factors interfered with our assessment of hypoalbuminemia. The most common causes of hypotension are significant blood loss and fluid depletion; however, in this case, the total blood loss was only 300 mL. Thus, in the context of persistent hypotension, we needed to consider other potential issues, including allergic reactions, pneumothorax, heart failure, and orthostatic hypotension: especially with further BP declines following position changes, and slight recovery in BP upon returning to the previous position. Common knowledge suggests that when a patient’s position changes from supine to head-down, gravity tends to direct blood toward the lower body, potentially reducing venous return and leading to decreased BP. We anticipated that the patient’s BP would recover after maintaining the position for some time. Unfortunately, as time passed and with fluid resuscitation, the patient’s BP continued to decrease.

Furthermore, the manifestations of coagulation dysfunction in this patient were not pronounced, which also affected our evaluation of hypoalbuminemia. Research indicates that hypoalbuminemia generally predisposes to a hypocoagulable state, particularly due to the lack of certain key clotting factors and plasma proteins (such as albumin), increasing the risk of bleeding.^[8]^ In contrast, this patient exhibited minimal intraoperative blood loss, making it difficult for the surgical team to identify significant oozing, which led to an oversight of coagulopathy until late in the procedure when laboratory tests revealed the patient had developed both hypoalbuminemia and coagulation dysfunction.

Reflecting on this case, we recognize some clinical signs that support the diagnosis of hypoalbuminemia. First, the patient had a large volume of ascites drained during surgery, which carried away a significant amount of protein. Second, we noted a decrease in the patient’s sensitivity to vasopressor medications as the surgery progressed. Theoretically, albumin carries many drug molecules within the bloodstream; therefore, lower plasma protein levels can reduce binding sites for these drugs, increasing the free (unbound) fraction and potentially affecting their efficacy and pharmacokinetic properties. Third, the patient presented with persistent severe hypotension, yet HR remained relatively stable, indicating that the body did not need to increase HR to improve oxygenation.

In summary, when encountering persistent refractory hypotension with minimal blood loss during surgery, it is essential to consider hypoalbuminemia after ruling out other causes. Timely testing of blood counts, coagulation profiles, and biochemical electrolytes, including serum albumin levels, is crucial. Guidelines recommend HSA supplementation for patients with significant ascites at a rate of 8 g of HSA per 1000 mL of ascitic fluid.^[9]^ Furthermore, preoperative assessments cannot fully predict potential intraoperative complications. Rapid physiological changes, such as decreased plasma colloid osmotic pressure, may occur during surgery; therefore, intraoperative monitoring and timely interventions are vital to maintaining hemodynamic stability.

## 4. Conclusion

Based on this case, we recommend that if an OC patient with hypertension experiences only mild bleeding and minimal HR variation but presents with refractory hypotension, even with normal preoperative biochemical tests (including serum albumin levels), we must still suspect hypoalbuminemia. Once confirmed through testing, HSA should be administered to alleviate hypovolemic shock symptoms. Additionally, caution is warranted regarding the potential for albumin use to exacerbate coagulation issues, which may necessitate plasma administration to address coagulopathy.

## Author contributions

**Writing – original draft:** Xinyan He, Hui Liu.

**Writing – review & editing:** Hui Liu.
